# A Life-Threating Postpartum Atypical Hemolytic-Uremic Syndrome with Multiorgan Involvement

**DOI:** 10.3390/jcm11236957

**Published:** 2022-11-25

**Authors:** Laura Sarno, Paolo Conca, Alfredo Capuano, Giovanni Tarantino, Domenico Russo, Maurizio Guida

**Affiliations:** 1 Department of Neurosciences, Reproductive Science and Dentistry, University Federico II, 80131 Naples, Italy; 2 Department of Clinical Medicine and Surgery, Regional Reference Center for Coagulation Disorders, University Federico II, 80131 Naples, Italy; 3 Department of Public Health, University Federico II, 80100 Naples, Italy; 4 Department of Clinical Medicine and Surgery, University Federico II, Via Pansini 5, 80131 Naples, Italy

**Keywords:** atypical Hemolytic Uremic Syndrome, postpartum, abruptio placenta, thrombotic microangiopathies

## Abstract

Atypical Hemolytic Uremic Syndrome is a very rare condition that can be triggered in predisposed patients. It can remain undiagnosed and can result in a life-threatening event or permanent renal failure. We report a case of a 36-year-old pregnant woman who developed atypical hemolytic uremic syndrome postpartum. She underwent an emergency caesarean section due to abruptio placenta, and she developed biochemical alterations suggestive of a thrombotic microangiopathy. Due to worsening of renal function after plasma exchange therapy, we decided to start therapy with eculizumab. Therapy was carried out with a weekly dose of 900 mg IV for five weeks. An improvement of clinical and biochemical parameters was rapidly observed, and her renal function completely recovered. The therapy was continued for six months, with a dose of 1200 mg of eculizumab every two weeks. One year after discontinuation of the therapy, her blood pressure and renal function were still normal. Our case confirms that it is important to promptly identify a pregnancy-related thrombotic microangiopathy and that early therapy can be life-saving for the patient and can preserve renal function, avoiding dialysis.

## 1. Introduction

Thrombotic microangiopathies (TMAs) are conditions characterized by thrombocytopenia, microangiopathic hemolytic anemia, and organ dysfunction, involving several districts, such as the brain, kidneys, eyes, heart, pancreas, liver, lungs, and skin. TMAs mainly include Hemolytic Uremic Syndromes (HUSs) and Thrombotic Thrombocytopenic Purpura (TTP) [1]. These conditions are both characterized by consumptive thrombocytopenia, mechanical hemolysis, and organ failure, but they have different underlying causes; they are classically associated with the thickening and inflammation of arterioles and capillaries, the detachment and swelling of endothelial cells, subendothelial widening, the accumulation of proteins and cellular debris, or platelet thrombi that block the vascular lumen [1,2,3,4]. Particularly, atypical HUS (aHUS) is characterized by the dysregulation of the complement system, leading to TMA, thrombosis, and organ dysfunction. Therefore, it might be triggered by conditions with increased complement activation, such as pregnancy, systemic lupus erythematosus, malignant hypertension, and hematopoietic stem cell transplantation [1,5,6]. It has been reported that up to 20% of cases of aHUS develop during pregnancy or postpartum [7]. Pregnancy-related TMAs are very rare, with a reported frequency of 1:25,000 births. They are obstetrical emergencies, and a prompt diagnosis and appropriate therapy are necessary to improve maternal and fetal outcomes [8]. Even if pregnancy-related TMAs occur very rarely, obstetricians are often the first specialist facing these emergencies, and it is important to highlight elements that could help in differential diagnosis.

We report a case of postpartum aHUS, highlighting difficulties that health-care providers encountered in the diagnosis and management of this case.

## 2. Case Report

A 36-year-old pregnant woman, I gravida, was admitted to the Mother and Child Department of University Hospital of Naples Federico II, at 31 + 4 weeks of gestation. She did not have any chronic conditions; her singleton pregnancy was spontaneously conceived, and she referred to us that the pregnancy was uneventful until the time of admission. She presented abdominal pain and heavy vaginal bleeding. Clinical examination revealed a fixed contraction of the uterus, increased heart rate (105 bpm), and low blood pressure (100/60 mmHg); diarrhea and fever were absent.

Combined blood tests presented alterations suggestive of TMA: thrombocytopenia (51,000/mL), anemia (hemoglobin 8.1 gr/dL), and signs of hemolysis (total bilirubine: 1.79 mg/dL [0.2–1.1]; LDH: 1100 U/L (227–450) and reticulocyte counts amplified. The haptoglobin consumption (undetectable [0.3–2.0 g/L]) and the presence of schistocytes in the blood smear confirmed the diagnosis.

The direct Coombs test was negative, D-dimer was slightly elevated (1.5 mg/L [0.1–0.6]), and plasma coagulation tests were within normal range. Increased serum creatinine (sCr: 2.3 mg/dL [0.6–1.1]) and elevated liver enzymes (AST: 127 U/L [0–34]) were evidenced.

Due to suspicions of an abruptio placenta, a prompt emergency caesarean section (CS) was performed, and the finding of a retro placental hematoma involving 2/3 of the placenta confirmed our suspicions. The newborn was born in cardiac arrest and was promptly reanimated by the neonatologist. The birth weight was 1350 g and the APGAR score was 0 at 1 min and 4 at 5 min.

**Figure 1:**
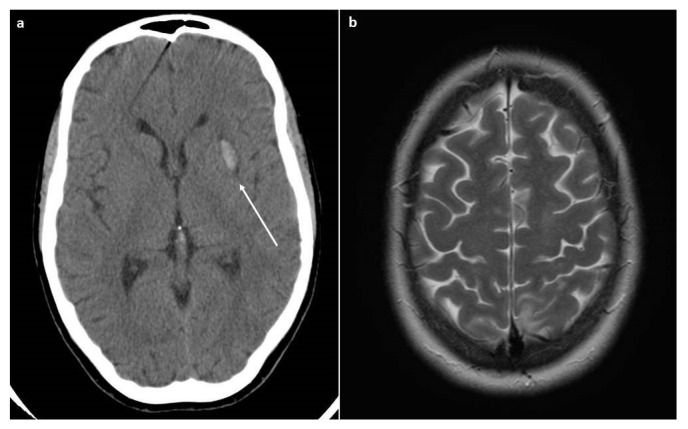
CT and MRI scans. (**a**) The CT scan revealed the presence of intracerebral hemorrhage (arrow). (**b**) The MRI scan (Axial T2-weighted image) revealed the presence of cerebral edema that is suggestive of PRES.

Soon after the CS, the patient underwent an eclamptic crisis (mean blood pressure value of 185/110 mmHg), and therapy with magnesium sulphate was started. She was admitted to the intensive care unit. A CT scan was performed due to the persistence of neurological symptoms (headache, scotomas, and confusion), and an intracerebral hemorrhage was detected, as seen in Figure 1.

The day after, the patient referred to us a new onset of headache and visual disturbance; therefore, an MRI was performed, showing vasogenic cerebral edema, specifically in the axial T2-weighted images, suggestive of posterior reversible encephalopathy syndrome (PRES).

A sudden worsening of thrombocytopenia and renal function was observed, and suspicions of pregnancy-related TMA were posed. ADAMTS-13 testing was not performed due to unavailability in our hospital, making it difficult to make the differential diagnosis between TTP and a-HUS. A single plasma exchange was carried out with fresh frozen plasma (FFP, 50 mL/kg), corresponding to the patient’s circulating plasma volume, without improvement of the clinical and biochemical status. C3 and C4 were normal. Factor H antibody levels were not tested due to unavailability.

Due to the worsening of renal function after plasma exchange, an empiric diagnosis of aHUS was made, and therapy with eculizumab (900 mg IV) was initiated four days after the CS. Therapy was carried out with a weekly dose of 900 mg IV for four weeks. Due to temporary unavailability, the patient received the anti-meningococcal A, B, C, W135, and Y vaccination before discharge only and was covered meanwhile by a six-week therapy with Ceftriaxone 2 gr daily until 14 days after the vaccination under strict clinical and laboratory monitoring.

**Figure 2:**
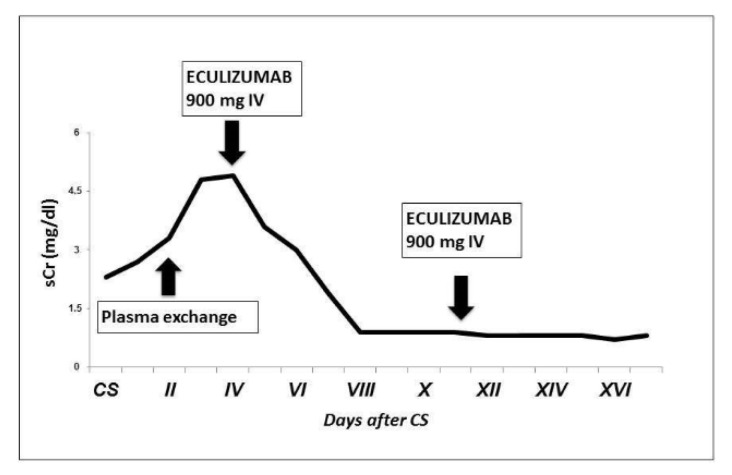
Patient’s creatinine trend after CS. We observed a progressive increase in sCr after CS and no improvement with plasma exchange; sCr started decreasing soon after the first dose of eculizumab.

**Figure 3:**
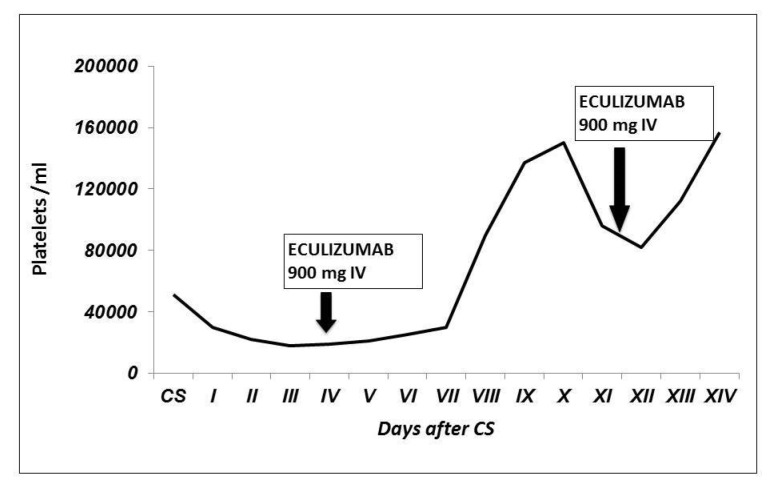
Patient’s platelet count trend after CS. We observed a progressive thrombocytopenia, not responsive to plasma exchange. The platelet count increased after eculizumab infusion, and it remained stable after the second injection.

An improvement of clinical and biochemical conditions was suddenly observed (Figure 2 and Figure 3).

The patient was discharged after the fifth injection.

At that time, her blood pressure was well controlled with methyldopa (500 mg twice a day), and the sCr and platelets count were normal (0.7 mg/dL and 224,000/mL, respectively). Proteinuria was 300 mg/24 h.

The therapy was continued for six months with a dose of 1200 mg of eculizumab every two weeks.

Genetic analysis (mutations in complement factor H, membrane cofactor protein, and complement factor I) revealed that she had no pathogenic sequence variants identified.

Three years after the discontinuation of the therapy, her blood pressure and renal function persisted normally.

## 3. Discussion

We reported a case of aHUS that developed in postpartum and was successfully treated with eculizumab.

HUS is classically divided into typical (shiga-toxin mediated or STEC) HUS and aHUS. It is characterized by a dysregulation of the alternative complement pathway, which is responsible for complement-mediated endothelial damage, leading to TMA and organ injuries [9].

Pregnancy is reported to be an important trigger of TMAs [10]. The first complication observed in our patient was abruptio placenta, as reported in a previous case of aHUS [11]. However, it is difficult to establish whether, in our case, aHUS developed as a complication of preeclampsia (as it has occasionally reported an overlap between these two conditions) or if the trigger was the pregnancy itself, the abruptio, or the CS [12]. Our case provides us with some point of discussion. Firstly, even if pregnancy-related TMAs are very rare events, with a reported incidence of around 1 in 25,000 pregnancies [11], all physicians should be aware of this possibility during pregnancy or postpartum. Indeed, when it occurs, it is very important to make a prompt diagnosis because an early start with the appropriate therapy is fundamental to save the patient and to avoid permanent renal failure. It has been estimated that, in case of TMAs, the early recognition and initiation of specific therapy substantially reduces the mortality rate to 10–20% [13]. Moreover, differentiating between TTP and aHUS is important because the therapy is completely different; in the first case, plasmapheresis is a lifesaving procedure, while it can be only temporarily or partially effective in aHUS, with no recovery of renal function in up to 80% of cases [14]. However, diagnosis can be very tricky because pregnancy itself can amplify the complement activity, and there are many conditions, such as hemolysis, elevated liver enzymes, and low platelets (HELLP) syndrome or preeclampsia, which are characterized by laboratoristic abnormalities of TMA, but they are pregnancy-related, and they generally recover after delivery [8]. We identified different elements that could help us in the diagnosis. Firstly, according to our experience, all cases that could imitate TMAs improved in 72 h postpartum. This was the only case that was characterized by a worsening of clinical and laboratoristic conditions in the postpartum period. Moreover, aHUS, just as it happened in our case, is more common in the postpartum period compared to other TMAs due to the loss of production of complement regulating proteins by the placenta [12]. It has been reported that up to 79% of aHUS develops in the postpartum period [11], while HELLP occurs more frequently during the third trimester and TTP during the second and third trimester. Finally, in aHUS, renal involvement is more severe compared to HELLP syndrome and TTP [8].

Moreover, ADAMTS-13 testing could have been very helpful in our case in order to formally exclude TTP, but it was not available in our hospital at that time. Indeed, we could avoid the use of plasmapheresis that could be an invasive procedure leading to several complications [12]. ADAMTS-13 is very important to differentiate between aHUS and TTP; ADAMTS-13 activity <10% is diagnostic of TTP, while it is above this value in cases of aHUS or HELLP syndrome. We strongly believe that a tertiary hospital should gear up for the activation of this test.

It has been suggested that screening for complement dysregulation includes routine complement measures, genotyping, and autoantibody testing [15]. In our case, C3 and C4 were normal; indeed, it has been reported that routine complement measures are not specific and have a low sensitivity [15]. Low serum C3 in pregnancy-related aHUS has been reported in around 39% of cases, and it seems more common (up to 60%) in patients with CFH or C3 variants. Other complement components, such as CFH, FI, or FB, had a low serum level in a lower percentage of cases (0–15%) [16]. Regarding genetic analysis, our patients had no pathogenic sequence variants identified. However, genetic analysis included only mutations in complement factor H, membrane cofactor protein, and complement factor I, while we did not perform other analyses. aHUS in pregnancy and postpartum has been associated with pathogenic sequence variants in 50–59% of the cases, mainly in complement factor H and complement factor I [16]. Factor H antibody testing has not been performed due to unavailability; it is recommended especially in case of the homozygous deletion of CFHR1 or CFHR3 [17]. However, a previous cohort study reported that the presence of factor H antibodies is not a common finding among pregnancy-related TMAs. In a series of 21 patients with pregnancy-related aHUS, no anti-factor H antibodies were detected in any of the patients [12]. These results give important information on the long-term prognosis and risk of relapse; indeed, patients with pathogenic complement gene variants or a high level of factor H autoantibodies are at an increased risk of TMA recurrance and long-term sequelae [15]. Even if these elements are important for the diagnosis and to orientate the prognosis, they are not available quickly enough; therefore, therapy is often empiric and based on clinical manifestation.

The overlap in the clinical and biochemical manifestation of pregnancy-related aHUS and preeclampsia or HELLP syndrome has been reported in previous case reports that are summarized in Table 1. In all these cases, the difficulties in differential diagnosis among these conditions were highlighted.

In Table 2, we report factors that helped in the differential diagnosis in our case.

Secondly, we think that the good outcome of the reported case was related to the early therapeutic decision to use eculizumab. Eculizumab is a humanized IgG2/4 kappa anti-C5 antibody that acts by blocking the enzymatic cleavage of C5 to C5a and C5b. Its safety and efficacy as therapy for aHUS have been established in previous prospective studies [11], and it has been approved for the treatment of aHUS and paroxysmal nocturnal hemoglobinuria. Prompt therapy with this antibody was associated with the normalization of renal function in other previous cases of pregnancy-related aHUS [11,25,27,28,29,30,31,32,33,34].

Thirdly, unfortunately, it is still uncertain how long the therapy should be prolonged with this antibody, and this is a challenge considering the high cost of eculizumab. We discontinued the therapy after six months, with the patient having normal renal function for more than three months, as suggested by Laurence [35], and our patient has not been had any relapse so far. However, prolongation of the therapy should be individualized, and discontinuation should be carefully monitored.

Finally, it is important to provide information to the patient regarding the risk of relapse in a subsequent pregnancy. A large French series reported that, even if the risk is higher in the first pregnancy, it remains relatively high in subsequent pregnancies, and the patient should be aware of such a risk [16]. However, evidence related to the safety of eculizumab in pregnancy [34] could give the chance of a good outcome in future pregnancies; indeed, many clinicians do not recommend against subsequent pregnancies [16]. However, further studies are needed to clarify this aspect.

## 4. Conclusions

Even if pregnancy-related TMAs are very rare events, it is important to train physicians to distinguish among different TMAs and to organize a multidisciplinary group including an obstetrician, nephrologist, hematologist, and anesthetist in order to optimize patients’ management.

## Figures and Tables

**Table 1:** Case reports of postpartum aHUS reporting overlap among aHUS, Preeclampsia (PE), and Hemolysis, Elevated Liver Enzymes Low Platelet Count (HELLP) syndrome.

Reference	Number of Pregnancy	Age	Gestational Age at Diagnosis of PE/HELLP	Diagnosis of aHUS	Genetic Analysis	Laboratoristic Findings	Treatment	Outcome
**Shanmugalingam et al.**, 2018 [18]	First pregnancy	27 ys	32 + 2 weeks	Postpartum, based on disproportionately impaired renal function, followed by renal biopsy	Normal	Factor I and H, ADMTS-13 activity, thrombophilic screen were normal. MCP/CD45 was reduced.	Eculizumab	Normal renal function two months postpartum
**Plante et al.**, 2002 [19]	First pregnancy	18 ys	38 weeks	Postpartum, based on deterioration in her mental status and acute kidney injury; renal biopsy 7 ws postpartum	N/A	N/A	Plasmapheresys	Hemodialysis For persistent renal failure
**Yamanaka et al.**, 2005 [20] **(CASE 1)**	Second pregnancy	28 ys	34 weeks	Postpartum, worsening of thrombocytopenia, and hemolysis	N/A	N/A	Plasma exchange	Hemodialysis for persistent renal failure
**Iannuzzi et al.**, 2006 [21]	Second pregnancy	37 ys	35 weeks	Postpartum, based on biochemical alterations	Evidence of a polymorfic variant of the HF1 gene (C-257T promoter region)	Thrombophilic screening, Factor H, ADAMTS-13 were normal	Plasma exchange, methylprednisolone ACE-inhibitor	Persistance of moderate renal insufficiency
**Zhou et al.**, 2012 [22]	First pregnancy	20 ys	34 weeks	Postpartum, based on clinical and biochemical conditions, followed by renal biopsy	N/A	N/A	Antihypertensive Aspirin Furosemide Methylprednisolone Intravanous immunoglobulin Plasma exchange Fresh frozen plasma	Hemodialysis for persistent renal failure
**Song et al.**, 2015 **(Case 1)** [23]	N/A	36 ys	39 weeks	Postpartum, based on clinical and biochemical conditions, followed by renal biopsy	CFH (p.Y402H) CFH (p.G936A)	Anti-CFH or CFI negative ADAMTS-13 normal	Plasma exchange Hemodialysis Prednisolone Methylprednisolone	End-stage renal disease
**Song et al.**, 2015 **(Case 2)** [23]	N/A	33 ys	39 weeks	Postpartum, based on clinical and biochemical conditions, followed by renal biopsy	CFH (p.V62I) CFH (p.G936A)	Anti-CFH or CFI negative ADAMTS-13 normal	Plasma exchange Hemodialysis Plasma infusion Prednisolone Methylprednisolone	Complete remission
**Kourouklaris et al.**, 2014 [24]	N/A	23 ys	31 weeks	Postpartum, based on clinical and biochemical conditions, followed by renal biopsy	N/A	ADAMTS-13 normal	Plasma exchange Hemodialysis Eculizumab	Remission
**Saad et al.**, 2016 [25]	Second pregnancy	19 ys	39 weeks	Postpartum worsening of laboratory investigations	Heterozygous carrier for a CD46 (MCP membrane cofactor protein) sequence variant (p.T383I; c.1148C > T)	ADAMTS 13 normal Low C3 and C4	Plasmapheresis Eculizumab	Remission
**Yamaguchi et al.**, 2016 [26]	First pregnancy	25 ys	37.5 weeks	Postpartum, mental state deterioration and worsening of laboratory investigations. Partial response to plasma exchange	homozygous mutation of the gene encoding complement factor H (pR1215G)	ADAMTS 13 a, C3, C4, CH50 were normal Negative factor H antibodies	Plasma exchange	Normal renal function at discharge

**Table 2:** Factors that helped in the differential diagnosis in our case [18,27].

	Preeclampsia/HELLP	TTP	aHUS
**Onset**	Third trimester (69%) Postpartum (31%) Rarely in second trimester	Second and third trimester	Postpartum (76%)
**Organ involvement**	Liver Nervous central system (NCS) Kidney	Nervous central system (NCS)	Kidney Rarely NCS (12%)
**Acute kidney injury**	Absent or mild	Absent or mild	Severe
**Postpartum evolution**	Improvement in 72 h	Absence of improvement	Absence of improvement/worsening

HELLP, Hemolysis, Elevated Liver Enzymes Low Platelet Count; TTP, Thrombotic Thrombocytopenic Purpura; aHUS, atypical Hemolytic Uremic Syndrome.

## Data Availability

Not applicable.
